# Ecological and environmental factors affecting the risk of tick-borne encephalitis in Europe, 2017 to 2021

**DOI:** 10.2807/1560-7917.ES.2023.28.42.2300121

**Published:** 2023-10-19

**Authors:** Francesca Dagostin, Valentina Tagliapietra, Giovanni Marini, Claudia Cataldo, Maria Bellenghi, Scilla Pizzarelli, Rosaria Rosanna Cammarano, William Wint, Neil S Alexander, Markus Neteler, Julia Haas, Timothée Dub, Luca Busani, Annapaola Rizzoli

**Affiliations:** 1 Research and Innovation Centre, Fondazione Edmund Mach, San Michele all'Adige (TN), Italy; 2 Centre for Gender-specific Medicine, Istituto Superiore di Sanità, Rome, Italy; 3 Knowledge Unit (Documentation, Library), Istituto Superiore di Sanità, Rome, Italy; 4 Environmental Research Group Oxford Ltd, Oxford, United Kingdom; 5 mundialis GmbH & Co. KG, Bonn, Germany; 6 Department of Health Security, Finnish Institute for Health and Welfare, Helsinki, Finland

**Keywords:** Europe, Tick-Borne Encephalitis, Vector-Borne Diseases, Incidence, Modelling, Covariates

## Abstract

**Background:**

Tick-borne encephalitis (TBE) is a disease which can lead to severe neurological symptoms, caused by the TBE virus (TBEV). The natural transmission cycle occurs in foci and involves ticks as vectors and several key hosts that act as reservoirs and amplifiers of the infection spread. Recently, the incidence of TBE in Europe has been rising in both endemic and new regions.

**Aim:**

In this study we want to provide comprehensive understanding of the main ecological and environmental factors that affect TBE spread across Europe.

**Methods:**

We searched available literature on covariates linked with the circulation of TBEV in Europe. We then assessed the best predictors for TBE incidence in 11 European countries by means of statistical regression, using data on human infections provided by the European Surveillance System (TESSy), averaged between 2017 and 2021.

**Results:**

We retrieved data from 62 full-text articles and identified 31 different covariates associated with TBE occurrence. Finally, we selected eight variables from the best model, including factors linked to vegetation cover, climate, and the presence of tick hosts.

**Discussion:**

The existing literature is heterogeneous, both in study design and covariate types. Here, we summarised and statistically validated the covariates affecting the variability of TBEV across Europe. The analysis of the factors enhancing disease emergence is a fundamental step towards the identification of potential hotspots of viral circulation. Hence, our results can support modelling efforts to estimate the risk of TBEV infections and help decision-makers implement surveillance and prevention campaigns.

**Key public health message**

**What did you want to address in this study?**

During the last decades, the number of tick-borne encephalitis (TBE) cases reported in Europe has increased, making TBE a growing concern for public health. It is difficult to identify TBE risk areas, as the circulation of the TBE virus depends on the interplay between numerous environmental and ecological conditions. Our aim was to summarise all the different aspects that enhance TBE spread and identify the main forces that affect the distribution of TBE human infections in Europe.

**What have we learnt from this study?**

TBE is a seasonal disease, dependent on tick abundance and activity. We found that TBE spread is favoured by the presence of key animal species, such as deer and rodents, in forested areas. We also discovered that specific climatic conditions, such as high precipitation during the driest months of the year, cold winters, small daily temperature variations and a steep decrease in late summer temperatures, increase the risk of TBE infections in humans.

**What are the implications of your findings for public health?**

The identification of all the environmental and ecological aspects that are influencing the risk of TBE across Europe is fundamental for the rapid assessment of potential TBE outbreaks. Hence, this study will be used to inform future risk mapping efforts in Europe and in the long run improve the targeting of prevention and control measures.

## Introduction

Tick-borne encephalitis (TBE) is a zoonotic disease which affects human and animal central nervous systems with mild to severe long-term sequelae, which may be fatal [1,2]. It is caused by the TBE virus (TBEV), a Flavivirus with currently three main subtypes and two additional subtypes recently proposed [3]. They circulate in nature among ticks, mostly those belonging to the *Ixodes ricinus* complex, and in several wildlife hosts. The three main subtypes circulating in the European Union and European Economic Area (EU/EEA) are the European (Eu), Siberian (Sib) and Far Eastern (FE) subtypes [3]. The European subtype TBEV-Eu, the most common one, is mainly associated with the biphasic form of TBE which has no chronic forms and presents symptoms with severe neurological sequelae in up to 10% of human cases and a fatality rate ranging from 1% to 2% [1]. Transmission to humans usually occurs after a tick bite, although food-borne infections after consumption of unpasteurised milk and dairy products from infected animals have been reported [4].

The geographical occurrence of TBEV is fragmented, with foci of infection (hotspots) that are difficult to identify and often vary in space and time [5]. Reporting of TBE cases in the EU/EEA is compulsory in 19 countries, voluntary in four (Belgium, France, Luxembourg and the Netherlands) and ‘not specified’ in one country (Croatia) [6], with 2,000 to 4,000 total cases reported yearly [7]. The European Centre for Disease Prevention and Control (ECDC) has reported increases in TBE incidence over the last years [6]. Major changes have been observed not only in the total number of reported cases, but also in the spatial distribution of the areas with active virus circulation, with the emergence of new TBEV foci in previously non-endemic countries [8-10].

The disease is preventable by vaccination along with personal protective measures which reduce the contact with infected ticks. The implementation of prevention and mitigation actions by public health authorities requires detailed knowledge of the disease’s distribution, which, in turn, needs a comprehensive understanding of the ecological factors driving the intensity of viral circulation and infection hazard.

In recent decades, growing attention has been devoted to assessing the factors driving TBEV circulation within the natural foci, with several studies aimed at identifying abiotic (e.g. [11-13]) or biotic (e.g. [5,14-17]) covariates. These include the analysis of the complex interactions between vectors and key vertebrate hosts that are strongly connected to the features of their local habitats. It is often difficult to establish the precise ecological conditions that favour TBE emergence and spread, a challenge that is reflected in the wide range of covariates that have been reported in the existing literature.

Our aim was therefore to obtain a more accurate understanding of the relationships occurring between a set of ecological variables and the incidence of TBE in humans across Europe, and to select those with highest impacts. As the TBE range has recently expanded and may continue to spread westward [8-10], northward [18-20] and to higher altitudes [21], we performed our analysis at a continental scale, responding to the need for a broader and more comprehensive understanding of the ecological forces driving such changes. This knowledge provides essential input to modern modelling approaches, based on quantitative disease data and a set of relevant covariates, which aim to predict the spatiotemporal risk of disease occurrence and its potential future spread.

## Methods

### Literature screening: search strategy and selection criteria

We performed a comprehensive literature search on TBE covariates following the principles of extending the PRISMA approach to scoping reviews [22]. Keywords were extracted from the MeSH database and EMBASE vocabulary, then integrated with text words found in relevant papers; see Supplementary Table S1 for the search strategy and keywords. The search was performed on 21 July 2020. We used the CAS STNext platform to search the MEDLINE, EMBASE, BIOSIS, SCISEARCH and CABA databases. We also searched SCOPUS (via Elsevier) by adapting the search strategy to the database-specific characteristics. We included in our review primary research studies (i.e. studies generating new data), modelling studies proposing quantitative analysis using explanatory variables and data collections with abstract and full-text documents available in English, published after 1 January 2000. We excluded studies with no data or with duplicated data (patents, editorials, letters, modelling studies with no data). We also excluded records with no denominator, no identified reference population, unavailable full-texts, and those that referred to data older than 2000 or were gathered outside the European Union and European Economic Area (EU/EEA). See Supplementary Figure S1 for the PRISMA flow diagram.

Four collaborators (AR, GM, LB, VT) independently evaluated potentially relevant records based on titles and abstracts. We then retrieved and read full texts of selected articles to assess their eligibility according to our inclusion and exclusion criteria and screened the references of selected publications to check for further sources of literature. We also added relevant articles published after the literature search was performed, up to 31 December 2021, by carrying out an additional search on PubMed. Finally, we used a pre-piloted data extraction spreadsheet to create our literature-based dataset and we selected covariates adopted in at least two articles for further analysis.

### Epidemiological data

We analysed TBE case-based data provided by the European Surveillance System (TESSy) and released by ECDC. Each record included the date of disease onset, the importation status and the most probable place of infection. Coded values for variables with geographical information followed the European nomenclature of territorial units for statistics (NUTS). When available, the probable place of infection was provided at the NUTS-3 level, corresponding to small regions for specific diagnosis, according to Regulation (EC) No 1059/2003 [23].

Of all the TBE cases recorded in TESSy, we included only the laboratory-confirmed cases reported from 1 January 2017 up to 31 December 2021, since most countries did not report the place of infection before 2017. Patients infected outside their country of residence or whose location of exposure was unknown or provided at low spatial resolution, were excluded. We included countries that reported at least 10 cases between 2017 and 2021 and notified the place of infection at NUTS-3 level for at least 75% of the cases. The countries selected according to these criteria were: Czechia, Finland, France, Germany, Hungary, Italy, Lithuania, Poland, Slovakia and Sweden. We also included data reported from Austria although at a lower spatial resolution (corresponding to NUTS-2 regions, i.e. basic regions for the application of regional policies) as the spatial extent of NUTS-2 units in Austria is comparable to the NUTS-3 regions of the other countries. For each region *i* we computed the average annual TBE incidence *Y_i_*, expressed as the number of cases per 100,000 inhabitants, over the period 2017 to 2021. The total population in each spatial unit was extracted using gridded population count datasets (100 m spatial resolution) provided by WorldPop [24].

### Covariate data

We collected raw data from various sources to compute the covariates identified through literature screening. The type of covariates considered were grouped into three different main categories, such as climatic, environmental and vertebrate host-related variables.

We used satellite images acquired by the moderate resolution imaging spectroradiometer and supplied by the National Aeronautics and Space Administration (NASA) with a resolution of 5.6 km as a source of land surface temperature and vegetation status as provided by the enhanced difference vegetation index (EVI). We downloaded the following products from the NASA Land Processes Distributed Active Archive Center: MOD11C1 Daily Land Surface Temperature and Emissivity [25], MOD11C3 Monthly Land and Surface Temperature and Emissivity [26] and MOD13C2 Vegetation Indices 16-Day [27]. We computed cumulative precipitation data from the European Centre for Medium-Range Weather Forecast’s fifth generation of European ReAnalysis (ERA5)-Land dataset and derived monthly time series of spatially enhanced relative humidity for Europe at 30 arc seconds resolution from ERA5-Land data [28]. We calculated bioclimatic predictors following the formulae stated in the World Climate database [29] and computed averaged autumnal cooling and spring warming rates from 2017 to 2021 by applying a linear regression to the average daily temperature against the Julian day in the period 1 August to 31 October and 1 February to 30 April, respectively [30].

We extracted proportions of land cover classes from the 2018 Corine Land Cover database, with a resolution of 0.25 km. We calculated the total length of forest roads from raster maps of road density (km road per km^2^), which were derived from linear road features extracted from OpenStreetMap datasets with a 1 km resolution. We derived estimates of snow and ice cover percentages from the 1 km consensus land-cover product [31]. Mean elevation was taken from the 1-km Global Multi-resolution Terrain Elevation dataset [32].

To account for the distribution of hosts across Europe, we used 1-km data about the probability of presence of selected critical reservoir species *(Apodemus flavicollis, Myodes glareolus)* and a single variable that describes the probability of presence of cervid species (*Dama dama*, *Cervus elaphus*, *Capreolus capreolus*) that have been reported to be the most important amplifier hosts for *I. ricinus* with respect to other ungulate species [33]. These variables were originally produced using spatial modelling techniques based on random forest and boosted regression trees [34,35].

We computed each covariate by averaging the raw values for the same spatial level as the available incidence data (NUTS-3 or NUTS-2). We also averaged covariate time series over the whole study period.

### Statistical analysis

Firstly, we performed single-variable analysis aimed at investigating the association between each covariate *x* and TBE incidence *Y_i_*. The response variable *Y_i_* was log-transformed before analysis to normalise the distribution [36], and we included a random effect on the reporting country to consider potential differences among national notification systems. We defined second-order linear mixed models, one for each explanatory variable *x*, of the form:


*log* (*Y_i_*) *= a_0_* + *a_1_ x_i_* + *a_2_ x_i_*
^2^ + *c* + *ε*

Where *a_0_*, *a_1_* and *a_2_* are the model coefficients, *c* is the random effect on the reporting country, and *x_i_* indicates the explanatory variable (Table 1). For each variable *x_i_* we tested both linear (L) and quadratic (Q) models. Quadratic models (Q) were selected as better models than linear (L) ones when the quadratic term proved to be significant (p value < 0.05). All explanatory variables, except those spanning an interval of 0–1 (EVI, land cover percentages, presence of hosts, rates of autumnal cooling and spring warming) were centred around their mean to avoid collinearity between linear and squared terms.

**Table 1:** Explanatory variables selected for statistical analysis of factors affecting the risk of tick-borne encephalitis, classified by data type^a^, EU/EEA, 2000–2021

Description of predictors (names)	Unit of measure	References of articles
**Climatic**		
Mean winter temperature (T_winter)	°C	[14,82]
Autumnal cooling rate (ac_rate)	Not applicable	[54,69]
Spring warming rate (sw_rate)	Not applicable	[48,71,83]
Annual mean temperature (BIO1^a^)	°C	[14,59-61,84,85]
Annual mean diurnal temperature range (BIO2^a^)	°C	[61,84]
Isothermality (BIO3^a^)	%	[60-62,84]
Temperature seasonality (BIO4^a^)	%	[61,84]
Minimum temperature of coldest month (BIO6^a^)	°C	[60-62]
Mean temperature of wettest quarter (BIO8^a^)	°C	[60-62]
Mean temperature of driest quarter (BIO9^a^)	°C	[59-62]
Mean temperature of warmest quarter (BIO10^a^)	°C	[60-62]
Mean temperature of coldest quarter (BIO11^a^)	°C	[60-62]
Annual total precipitation (BIO12^a^)	mm	[17,49,60-62,84]
Precipitation seasonality (BIO15^a^)	%	[60-62,84]
Total precipitation of wettest quarter (BIO16^a^)	mm	[60-62]
Total precipitation of driest quarter (BIO17^a^)	mm	[60-62]
Total precipitation of warmest quarter (BIO18^a^)	mm	[60-62]
Total precipitation of coldest quarter (BIO19^a^)	mm	[60-62]
Annual mean relative humidity (RH)	%	[59,71]
Mean saturation deficit (SD)	mmHg	[48,50,86]
**Environmental**		
Mean elevation (Elev)	m a.s.l.	[60,69,84,87]
Percentage of forested area (CLC_31)	%	[49,55,59-61,63,71-76,84,87,88]
Percentage of area with low vegetation (CLC_32)	%	[49,61,84]
Percentage of agricultural land (CLC_2)	%	[13,59,73,76,84]
Percentage of urban area (CLC_1)	%	[59,75,84]
Percentage of area covered by snow (SnowIce)	%	[75,86]
Length of forest roads (For_length)	km	[63,88]
Enhanced difference vegetation index (EVI)	Not applicable	[49,61,75,87,89]
**Vertebrate hosts**		
Cervids (*Capreolus capreolus, Cervus elaphus, Dama dama*) probability of presence (host_cervids)	Not applicable	[15-17,54,56,57,63-65,71]
Rodent (*Apodemus flavicollis*) probability of presence (host_af)	Not applicable	[15, 51,54,83,85]
Rodent (*Myodes glareolus*) probability of presence (host_mg)	Not applicable	[15,51,54,58,83,85,90]

CLC: Corine land cover; EU/EEA: European Union and European Economic Area.

^a^ Bioclimatic predictors are named following the WordClim coding. Predictors’ names are reported in brackets.

Afterwards, we used multiple linear regression to select the explanatory variables with the highest predictive power for TBE incidence (multivariable analysis). We built a full model considering all covariates with a significant (p value < 0.05) coefficient *a_1_* in the models previously described. For this subset, quadratic terms (coefficient *a_2_*) were also included if significant in the single-variable analysis. All selected variables were examined for multicollinearity by computing Pearson’s r pairwise correlation coefficients and variance inflation factors [37]. Among highly correlated variables, we kept the ones with lowest Akaike information criterion (AIC) in single-variable analysis. We then computed all possible submodels and ranked them according to their AIC score. We finally selected the best parsimonious model with lowest AIC among a set of candidates with approximately equal performances (Δ*AIC* < 2) [38]. Model assumptions were verified by checking the model’s residuals for any pattern or dependency [39]. We obtained p values according to the Satterthwaite method [40]. All analyses were carried out using R v.4.1.2 [41] and packages dplyr [42], exactextractr [43], raster [44], lme4 [45], lmerTest [46] and MuMIn [47].

## Results

### Literature screening

**Figure 1:**
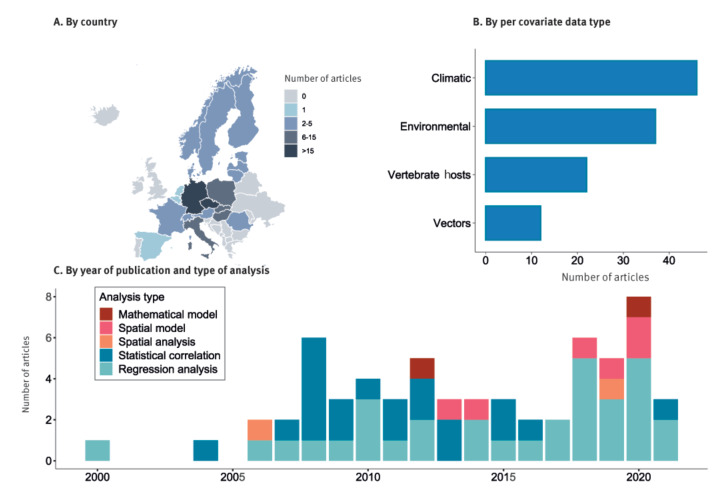
Main characteristics of included studies on factors affecting the risk of tick-borne encephalitis, EU/EEA, 2000–2021 (n = 62)

After applying our selection criteria, we retrieved relevant information from 62 full-text articles (see Supplementary Figure S1 and Table S2 for the PRISMA flow diagram and list of articles). Most studies focused on central-eastern countries, such as Germany (16 studies) and Czechia (18 studies) (Figure 1A). The types of covariates considered were predominantly related to climate (46 studies), environment (37 studies) and competent and incompetent hosts (22 studies). We also included articles considering vector–host related data (12 studies), but as we selected covariates that were adopted in at least two articles for further analysis, none of the parameters used in such studies met this criterion (Figure 1B). The methodological approaches ranged from local surveys to more complex large-scale spatial models aimed at TBE risk assessment (Figure 1C).

EU/EEA: European Union and European Economic Area.

We selected thirty-one covariates adopted in at least two articles for further analysis (Table 1).

### Epidemiological and statistical analysis

**Figure 2:**
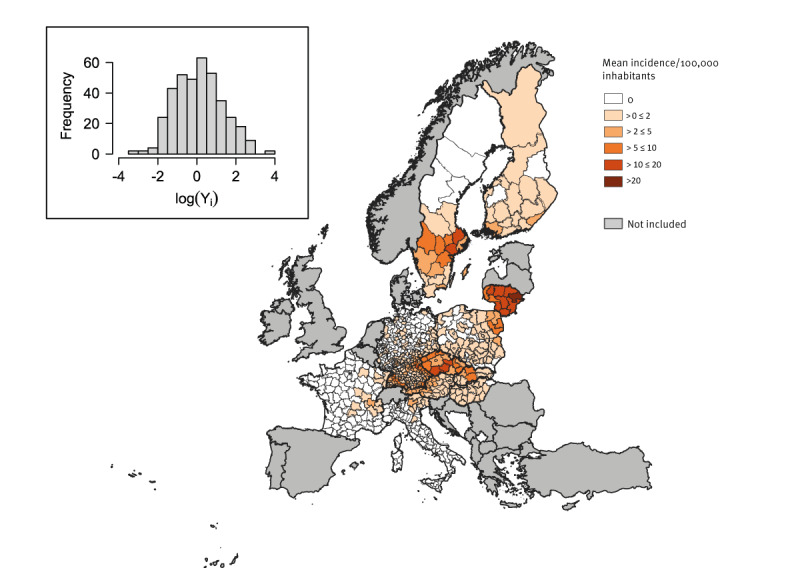
Mean tick-borne encephalitis incidence in the 380 NUTS regions selected for analysis, EU/EEA, 2017–2021

In the period 2017 to 2021 a total of 12,289 confirmed cases with known place of infection were reported to ECDC from 371 NUTS-3 and nine NUTS-2 European regions from the 11 countries included in the study. The 4-year mean incidence across the considered NUTS ranged between 0.04 and 45.66, with an average (of all mean values) of 3.74 per 100,000 inhabitants (Figure 2).

EU/EEA: European Union and European Economic Area; TBE: tick-borne encephalitis.

Inset: distribution of the log-transformed TBE incidence (log(Y_i_)).

The single-variable analysis proved that the distribution of the mean log-transformed TBE incidence transmission in Europe was significantly related to almost all factors, except for the total length of forest roads (For_length) (Table 2).

**Table 2:** Results of the single-variable analysis on factors affecting the risk of tick-borne encephalitis, ordered by AIC, EU/EEA, 2000–2021

Predictors	Best model type	*a_0_*	p value (*a_0_*)	*a_1_*	p value (*a_1_*)	*a_2_*	p value (*a_2_*)	R^2^ m	R^2^ c	AIC
T_winter	Q	0.27	0.34	**−0.23**	**< 0.001**	**−0.01**	**< 0.001**	0.23	0.61	995.29
Elev	Q	0.36	0.40	**2.31**	**< 0.001**	**−1.18**	**< 0.001**	0.09	0.75	1,001.24
SD	L	0.14	0.63	**−.77**	**< 0.001**	Not applicable		0.18	0.61	1,004·07
BIO11	Q	0.28	0.32	**−0.24**	**< 0.001**	**−0.02**	**< 0.001**	0.18	0.58	1,010.20
BIO6	Q	0.30	0.31	**−0.17**	**< 0.001**	**−0.01**	**< 0.001**	0.13	0.59	1,015.75
BIO10	Q	0.44	0.17	**−0.21**	**< 0.001**	**−0.03**	**< 0.001**	0.13	0.59	1,018.76
CLC_31	L	−0.66	0.10	**2.43**	**< 0.001**	Not applicable		0.07	0.65	1,029.83
BIO17	Q	0.34	0.41	**0.01**	**< 0.001**	**0.00003**	**0.01**	0.05	0.69	1,031.70
RH	Q	0.51	0.14	**0.14**	**< 0.001**	**−0.04**	**< 0.001**	0.09	0.61	1,036.87
BIO12	Q	0.31	0.47	**0.002**	**< 0.001**	**−0.000001**	**0.04**	0.04	0.71	1,037.94
CLC_1	L	0.43	0.25	**−2.15**	**< 0.001**	Not applicable		0.04	0.64	1,039.15
BIO18	Q	0.27	0.49	**0.01**	**< 0.001**	**−0.000001**	**0.001**	0.04	0.68	1,040.89
BIO1	Q	0.32	0.33	**−0.13**	**< 0.001**	**−0.008**	**< 0.001**	0.07	0.58	1,046.00
BIO16	L	0.23	0.60	**0.004**	**< 0.001**	Not applicable		0.03	0.70	1,047.13
BIO19	L	0.32	0.43	**0.005**	**< 0.001**	Not applicable		0.03	0.68	1,050.77
host_cervids	Q	**−2.70**	**< 0.001**	**11.72**	**< 0.001**	**−10.68**	**< 0.001**	0.04	0.67	1,055.19
BIO9	Q	0.24	0.51	**−0.04**	**< 0.001**	**−0.003**	**0.04**	0.03	0.60	1,058.42
ac_rate	Q	**−12.88**	**0.001**	**−153.39**	**< 0.001**	**-440.51**	**< 0.001**	0.03	0.63	1,061.64
sw_rate	L	**−1.86**	**0.01**	**10.41**	**< 0.001**	Not applicable		0.03	0.59	1,061.94
host_af	L	**−**0.79	0.09	**1.76**	**< 0.001**	Not applicable		0.04	0.62	1,062.34
BIO3	Q	**−**0.001	0.99	**−0.08**	**0.04**	**0.04**	**< 0.001**	0.03	0.61	1,064.14
CLC_2	Q	**−**0.35	0.43	**3.70**	**0.001**	**−4.35**	**< 0.001**	0.01	0.62	1,064.42
host_mg	Q	**−2.31**	**0.02**	**8.95**	**0.003**	**−7.40**	**0.001**	0.02	0.62	1,065.69
BIO15	L	0.21	0.56	**−0.02**	**0.004**	Not applicable		0.01	0.60	1,066.50
CLC_32	L	0.02	0.95	**4.36**	**0.01**	Not applicable		0.01	0.63	1,067.48
EVI	L	−1.12	0.09	**4.19**	**0.01**	Not applicable		0.01	0.63	1,068.05
BIO4	L	0.18	0.60	**0.8**	**0.02**	Not applicable		0.02	0.59	1,068.84
BIO2	L	0.21	0.56	**−0.13**	**0.02**	Not applicable		0.01	0.61	1,069.33
SnowIce	L	0.19	0.61	**55.52**	**0.03**	Not applicable		0.01	0.63	1,069.93
BIO8	L	0.27	0.46	**−0.03**	**0.03**	Not applicable		0.01	0.61	1,070.12
For_length	L	0.21	0.57	0.0002	0.56	Not applicable		0.01	0.61	1,074.28

*a_0_*: intercept; *a_1_*: coefficient of linear term; *a_2_*: coefficient of quadratic term; AIC: Akaike information criterion; CLC: Corine land cover; EU/EEA: European Union and European Economic Area; L: linear model; Q: quadratic model; R^2^m: marginal R^2^; R^2^c: conditional R^2^.

Significant coefficients are presented in bold. See Table 1 for a description of the predictors.

After checking for pairwise correlations (see Supplementary Figure S2 for the correlation matrix of covariates), we kept 22 covariates for multivariable analysis (see Supplementary Table S3 for the list of candidate models). Finally, eight covariates were selected in the best parsimonious model (Table 3). The model was characterised by a reasonably good fit (marginal R^2^ = 0.28, conditional R^2^ = 0.66, AIC = 921.56), higher than any of the fits obtained in single-variable analysis. Visual inspection of residual plots did not reveal any obvious deviations from normality.

**Table 3:** Results of multi-variable analysis of factors affecting the risk of tick-borne encephalitis, EU/EEA, 2000–2021

Description of predictors (names)	Predictors	Coefficient	95% CI	t value	p value
Model intercept	Intercept	−4.83	−5.9 to −3.76	−4.50	< 0.001
% of forest cover in the area	CLC_31	0.89	0.48 to 1.29	2.20	0.03
Autumnal cooling rate	ac_rate	−10.93	−14.3 to −7.57	−3.25	0.001
Mean winter temperature	T_winter	−0.19	−0.22 to −0.17	−7.59	< 0.001
Total precipitation of the driest quarter	BIO17	0.005	−0.31 to −0.19	4.08	< 0.001
Mean diurnal temperature range	BIO2	−0.25	0.004 to 0.006	−4.16	< 0.001
Probability of presence of *Apodemus flavicollis*	host_af	2.04	1.51 to 2.57	3.83	< 0.001
Probability of presence of *Myodes glareolus*	host_mg	−1.89	−2.44 to −1.34	−3.41	< 0.001
Probability of presence of cervids	host_cervids	9.77	7.55 to 11.98	4.41	< 0.001
Squared probability of presence of cervids	host_cervids^2^	−8.89	−10.86 to −6.93	−4.52	< 0.001

CI: confidence interval; EU/EEA: European Union and European Economic Area.

Estimated regression coefficients, 95% confidence intervals, t values, and p values for the best parsimonious model. Standard deviation of the random effect for ‘Country’ = 0.80. Observations = 380. Countries = 11. Marginal R^2^ = 0.28, conditional R^2^ = 0.66. AIC = 921.56.

**Figure 3:**
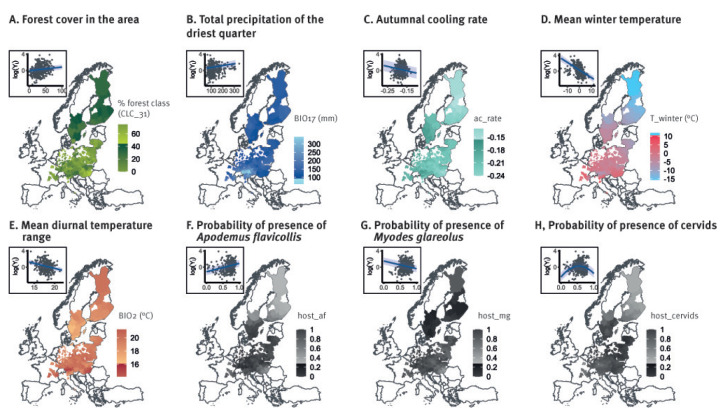
Best model conditional predictions of factors affecting the risk of tick-borne encephalitis, EU/EEA, 2000–2021

To better grasp how each predictor selected in the best model was related to the distribution of human TBE incidence in Europe, we computed conditional predictions for the log-transformed TBE incidence (log(*Y_i_*)) (Figure 3); all variables were kept at their average value, except for the one shown in each specific graph.

EU/EEA: European Union and European Economic Area.

Line: average, blue area: 95% confidence interval, dots: observed data. Actual covariate values are shown in the maps.

Our results show that higher TBE incidence in humans was linked to higher percentages of forested area and high precipitation in the driest quarter. Higher rates of autumnal cooling, a steep decrease in late summer temperatures, colder winters and smaller variations in daily temperatures values were also related to higher TBE incidence. Critical hosts species appear to have different impacts: disease incidence increased with the probability of presence of *A. flavicollis*, while it decreased in areas characterised by the presence of *M. glareolus*. We found a parabolic relationship between human incidence and the probability of presence of cervids (*C. capreolus*, *D. dama*, *C. elaphus*) (Figure 3).

## Discussion

Tick-borne encephalitis is an increasing concern for European public health. The risk of infection depends on the co-occurrence of a set of ecological factors that have not been completely identified yet. Our literature screening revealed substantial heterogeneity in the selected studies. This diversity depends on the different goals of the studies, mostly focused on local investigations of TBEV in ticks [21,48-53] and hosts [5^4^-^58^]. Broader modelling studies assessing the geographical distribution of the pathogen are rarer and usually based on climatic predictors [59-62]. Overall, we identified 31 covariates, and the single-variable analysis proved how TBE incidence was significantly affected by almost all of them. This result is in accordance with the available literature and provides additional confirmation of previous published analyses. Assessing the drivers shaping disease distribution is a fundamental step needed to successfully model disease risk. Eight factors proved to be the most effective for explaining the distribution of TBE incidence in Europe.

Firstly, it is essential to consider the presence of competent and non-competent tick-feeding hosts and the features of their habitat. Competent TBEV reservoir hosts are mainly small rodents and insectivores that support both virus circulation and feeding ticks, while non-competent hosts act as amplifiers of the vector population. All host-related variables were selected as relevant predictors in the best parsimonious model, underlining the importance of considering the presence of critical species when modelling the risk of emergence of new TBE hotspots. This is usually accomplished using local game animal density data as a proxy for host density [16,17,63-66]. However, it is difficult to retrieve such standardised information at the European scale, and these assessments are generally focused on non-competent hosts. In this study we used the probability of presence of rodents and cervids and validated their impact on the distribution of TBE incidence. These datasets could, therefore, serve as good predictors in future studies aimed at assessing the risk of disease outbreaks in vast geographical areas.

Rodents such as *A. flavicollis* and *M. glareolus*, the most common and widespread species inhabiting, often sympatrically, forested areas, play a pivotal role in the enzootic cycle of TBEV. They are well known susceptible hosts capable of transmitting the virus to the feeding ticks both systemically, developing viraemia, and non-systemically, via co-feeding [67]. Interestingly, we found a positive relationship between TBE incidence and *A. flavicollis*, but a negative one with *M. glareolus*. One possible explanation could be the fact that *M. glareolus* acquires resistance to tick infestation [68], therefore hampering the co-feeding mechanism which is allegedly the most efficient mechanism contributing to TBEV circulation.

In addition to rodents, ungulates also play a major role in TBEV epidemiology [15,17,51,69,70] as they are able to amplify tick abundance by acting as hosts to adult stages and by moving them over long distances. At the same time, as non-competent hosts, they can divert tick bites from competent hosts (dilution effect), causing a decrease of TBEV prevalence in ticks after a certain threshold density is reached [15,51]. Our results confirm this statement, as TBE incidence is lower in the regions characterised by low probability of presence of cervid species, then increases to reach a peak and finally decreases again in areas where the probability of occurrence is at its maximum.

The total proportion of forested areas (such as broad-leaved, coniferous and mixed forest) was also found to be a good predictor for TBE incidence, with a positive impact on disease occurrence in humans. Forest areas provide suitable habitat and resources for ungulates, rodents and ticks, thus promoting their encounter rate and boosting the risk of occurrence of human TBE cases [55,71-76]. Moreover, human activity and behaviour can act in synergy with ecological and environmental factors by increasing the chances of exposure to infected ticks, as people engaged in recreational or occupational activities in forests are at increased risk of tick encounters and bites [77,78]. The time spent in mixed forest for recreational purposes (of ≥ 10 h/week) has been positively associated with an increased TBE risk, and so were other activities such as harvesting forest foods and being employed as a forester or non-specialised worker [77].

Tick-borne encephalitis is a seasonal disease, dependent on tick abundance and activity, which in turn is strongly affected by climatic conditions. We tested several variables related to temperature, precipitation and relative humidity in the full model and found four factors as the best predictors, namely the rate of autumnal cooling, the mean temperature registered in winter, the mean diurnal temperature range, and the total precipitation of the driest quarter.

At the continental scale, areas characterised by rapid temperature drops in late summer and early autumn are generally affected by higher values of TBE incidence, while at the local scale, the impact of daily temperature variations on the prevalence of TBEV in ticks based on field data showed contrasting results. For example, there was no evidence of any effect of a rapid autumnal temperature decrease on the minimum infection rate of nymphs in the following spring in a TBE focus in Germany [79]. Such results were obtained by computing the decadal mean daily maximum air temperature in spring and autumn. On the other hand, the autumnal cooling rate (computed as in Randolph et al. [30]) proved to be a crucial ecological driver for co-feeding transmission of TBEV and for the maintenance of a TBE hotspot in northern Italy [54,69]. Autumnal cooling plays a key role in TBEV epidemiology [47] as a steep decrease in late summer temperatures induces a behavioural diapause that favours a synchronous larval and nymphal activity the following spring, an event that is generally considered one of the most critical factors in TBEV transmission [30,54,69].

We hypothesise that the positive correlation between high TBE incidence and low winter temperature could be biased by the high incidence of cases registered between 2017 and 2021 in countries that exhibit low temperatures in winter, such as Austria, Czechia, Finland, Lithuania and Sweden. From an ecological perspective, this result can be explained by the fact that cold winter temperatures induce diapause in ixodid ticks, sheltering them from unfavourable climatic conditions and supporting their overwintering survival [80]. On the other hand, TBE incidence decreases in regions characterised by strong daily temperature variations. Such changes in temperature may affect questing behaviour of ticks and thus the probability of contact with hosts and their survival [11,50]. The total precipitation of the driest quarter is another key indirect factor that can influence tick behaviour and survival. Hence, higher precipitation might lead to lower tick mortality and continued tick questing during the driest months of the year, but also ensure that ticks in shelters survive to later activity periods [81].

## Conclusion

TBEV distribution is shaped by the interplay of multiple climatic, environmental and ecological factors that exert a crucial role in the life cycle of ticks and TBEV circulation. Through our approach, we provided insights into the combination of covariates that appear to be crucial in affecting TBEV occurrence, defined their main data sources and established their interrelation with human TBE incidence at a large scale, considering the countries that notified TBE cases to ECDC at the highest possible spatial detail. The early identification of potential health threats derived from TBEV circulation is fundamental to improve timely detection and awareness of infectious disease events at the earliest stage of their emergence. Hence, this study could inform future modelling efforts aimed at assessing TBE risk across Europe and support competent authorities in deploying One Health integrated actions in existing and new potential risk areas.

## Disclaimer

The views and opinions of the authors expressed herein do not necessarily state or reflect those of ECDC. The accuracy of the authors’ statistical analysis and the findings they report are not the responsibility of ECDC. ECDC is not responsible for conclusions or opinions drawn from the data provided. ECDC is not responsible for the correctness of the data and for data management, data merging and data collation after provision of the data. ECDC shall not be held liable for improper or incorrect use of the data.

## Supplementary Data

352000 Supplement
