# Supplementary material for: Ecological and environmental factors affecting the risk of tick-borne encephalitis in Europe, 2017 to 2021
Source: Euro Surveill. 2023 Oct 19;28(42):2300121. doi: 10.2807/1560-7917.ES.2023.28.42.2300121 (PMC10588310; doi:10.2807/1560-7917.ES.2023.28.42.2300121)
Supplement: Supplement [file 23-00121_DAGOSTIN_Supplement.pdf]

SUPPLEMENTARY MATERIAL

This supplementary material is hosted by Eurosurveillance as supporting information alongside the article "Ecological and environmental factors affecting the risk of tick-borne encephalitis in Europe", on behalf of the authors, who remain responsible for the accuracy and appropriateness of the content. The same standards for ethics, copyright, attributions and permissions as for the article apply. Supplements are not edited by Eurosurveillance and the journal is not responsible for the maintenance of any links or email addresses provided therein.

Search strategy and keywords used for literature screening

Search Identifier	Search terms
L1	ENCEPHALITIS, TICK-BORNE/CT
L2	ENCEPHALITIS TICK BORNE/TI,AB
L3	TICK BORNE ENCEPHALITIS/TI,AB
L4	ENCEPHALITIS RUSSIAN SPRING-SUMMER/TI,AB
L5	SPRING SUMMER ENCEPHALITIS RUSSIAN/TI,AB
L6	RUSSIAN SPRING SUMMER ENCEPHALITIS/TI,AB
L7	ENCEPHALITIS FAR EASTERN RUSSIAN/TI,AB
L8	FAR EASTERN RUSSIAN ENCEPHALITIS/TI,AB
L9	ENCEPHALITIS EUROPEAN TICK BORNE/TI,AB
L10	TICK BORNE ENCEPHALITIS EUROPEAN/TI,AB
L11	EUROPEAN TICK BORNE ENCEPHALITIS/TI,AB
L12	ENCEPHALITIS CENTRAL EUROPEAN/TI,AB
L13	CENTRAL EUROPEAN ENCEPHALITIS/TI,AB
L14	ENCEPHALITIS VIRUSES, TICK-BORNE/CT
L15	ENCEPHALITIS VIRUS## TICK BORNE/TI,AB
L16	VIRUS## TICK BORNE ENCEPHALITIS/TI,AB
L17	TICK BORNE ENCEPHALITIS VIRUS##/TI,AB
L18	TBEV/TI,AB
L19	TBE/TI,AB
L20	L1-L19
L21	TEMPERATURE/BI
L22	HUMIDITY/BI
L23	WIND SPEED/BI
L24	WIND/BI
L25	CLIMATE FACTOR#/BI OR CLIMATE CHANGE/BI
L26	CLIMATE/BI
L27	WEATHER/CT
L28	WEATHER/TI,AB
L29	AIR MOVEMENTS/BI OR RAIN/BI
L30	METEOROLOGICAL FACTOR#/BI
L31	RAINFALL/BI
L32	HEAT OR COLD
L33	SEASON?
L34	L20 AND L21-L33
L35	ANIMALS, WILD/CT
L36	WILD ANIM AL/CT
L37	WILD (S) ANIMAL#/TI,AB
L38	NONDOMESTIC? (S) ANIMAL#/TI,AB
L39	NON DOMESTIC? (S) ANIMAL#/TI,AB
L40	NON DOMESTIC? (S) FAUNA/TI,AB
L41	NONDOMESTIC? (S) FAUNA/TI,AB
L42	STRAY (S) ANIMAL#/TI,AB
L43	FERAL (S) ANIMAL#/TI,AB
L44	ARTHROPODS/CT,TI,AB

L45	ARTHROPOD/CT,TI,AB
L46	TICKS+NT/CT
L47	TICK+NT/CT
L48	TICK#/TI,AB
L49	CULICIDAE/BI OR MOSQUITO?/TI,AB
L50	MOSQUITO+NT/CT
L51	DIPTERA/CT,TI,AB
L52	FLY/TI,AB OR FLIES/TI,AB
L53	(HUMAN OR HUMANS OR MAN OR MEN OR WOMAN OR WOMEN)/BI
L54	(BOY# OR GIRL# OR CHILD? OR INFAN? OR ADULT# OR PATIENT# OR ADOLESCEN?)/BI
L55	VITRO/CT,TI,AB
L56	L34 AND L35-L55
L57	L56 NOT (CASE REPORTS/CT OR CASE REPORT/CT OR CASE REPORT#/TI,AB)
L58	DUP REM L57 (1057 DUPLICATES REMOVED)
L59	L58 NOT (ARABIC/LA OR RUSSIAN/LA OR CHINESE/LA OR JAPANESE/LA OR TURKISH/LA)
L60	L59 NOT (EDITORIAL/DT OR NEW/DT OR LETTER/DT OR COMMENT/DT)
L61	L60 AND 1990-2020/PY

Table S1 – Search strategy and keywords used for literature screening.

LEGEND

Field codes:

AB=Abstract
BI=Basic Index (equivalent of All Fields)
CT=Controlled Term
DT=Document Type (equivalent of Type of Publication)
LA=Language
PY=Publication Year
TI=Title

Proximity Operator:

(S)= Terms must occur within the same "sentence" or subfield

Truncation and Character Masking Symbols:

? = Any number of characters to the right of the term
#= One or zero characters at the designated position

PRISMA flow diagram for the systematic literature screening

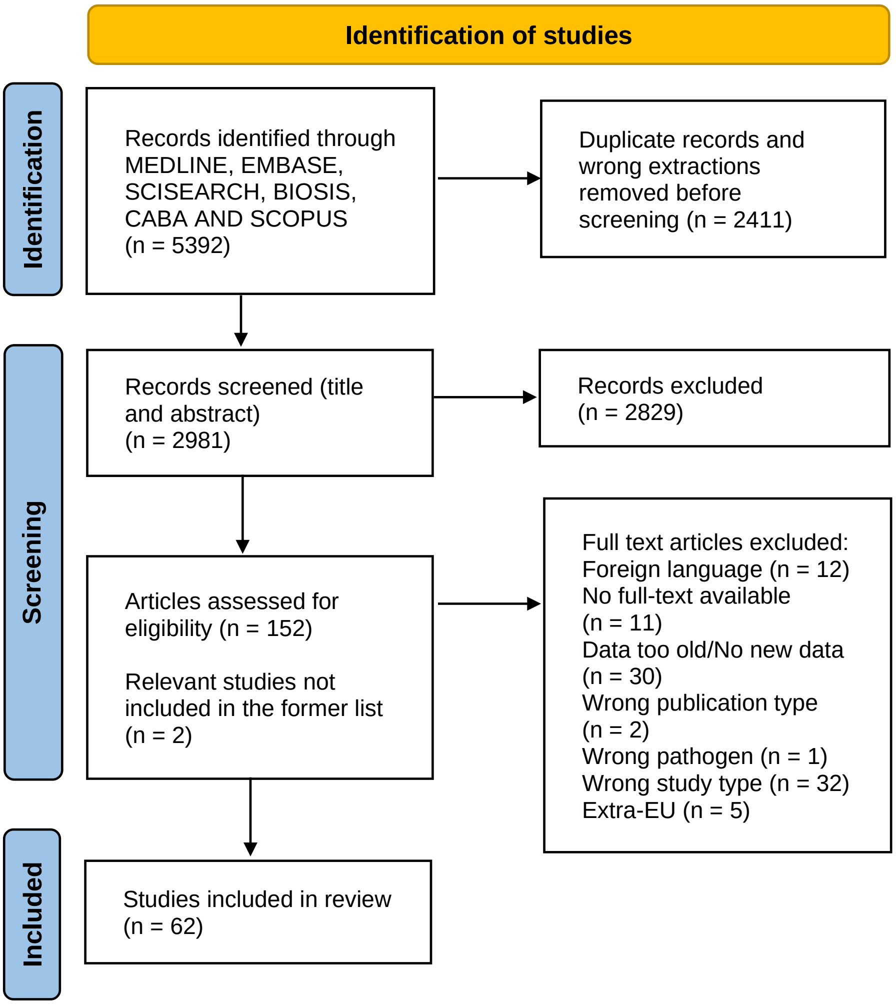

Figure S1 – PRISMA flow diagram for the systematic literature screening detailing the database searches, the number of abstracts screened, and the full texts retrieved.

Full references of the selected articles

Andreassen, A., Jore, S., Cuber, P., Dudman, S., Tengs, T., Isaksen, K., Hygen, H.O., Viljugrein, H., Ånestad, G., Ottesen, P., Vainio, K., 2012. Prevalence of tick borne encephalitis virus in tick nymphs in relation to climatic factors on the southern coast of Norway. Parasit. Vectors 5, 177. https://doi.org/10.1186/1756-3305-5-177

Barandika, J.F., Hurtado, A., Juste, R.A., García-Pérez, A.L., 2010. Seasonal Dynamics of *Ixodes ricinus* in a 3-Year Period in Northern Spain: First Survey on the Presence of Tick-Borne Encephalitis Virus. Vector-Borne Zoonotic Dis. 10, 1027–1035. https://doi.org/10.1089/vbz.2009.0148

Bartosik, K., Lachowska-Kotowska, P., Szymańska, J., Wójcik-Fatla, A., Pabis, A., Buczek, A., 2011. Environmental conditioning of incidence of tick-borne encephalitis in the south-eastern Poland in 1996-2006. Ann. Agric. Environ. Med. AAEM 18, 119–126.

Bolzoni, L., Rosà, R., Cagnacci, F., Rizzoli, A., 2012. Effect of deer density on tick infestation of rodents and the hazard of tick-borne encephalitis. II: Population and infection models. Int. J. Parasitol. 42, 373–381. https://doi.org/10.1016/j.ijpara.2012.02.006

Bona, M., Blaňárová, L., Stanko, M., Mošanský, L., Čepčeková, E., Víchová, B., 2021. Impact of climate factors on the seasonal activity of ticks and temporal dynamics of tick-borne pathogens in an area with a large tick species diversity in Slovakia, Central Europe. Biologia (Bratisl.). https://doi.org/10.1007/s11756-021-00902-x

Borde, J.P., Kaier, K., Hehn, P., Matzarakis, A., Frey, S., Bestehorn, M., Dobler, G., Chitimia-Dobler, L., 2021. The complex interplay of climate, TBEV vector dynamics and TBEV infection rates in ticks—Monitoring a natural TBEV focus in Germany, 2009–2018. PLOS ONE 16, e0244668. https://doi.org/10.1371/journal.pone.0244668

Bournez, L., Umhang, G., Moinet, M., Richomme, C., Demerson, J.-M., Caillot, C., Devillers, E., Boucher, J.-M., Hansmann, Y., Boué, F., Moutailler, S., 2020. Tick-Borne Encephalitis Virus: Seasonal and Annual Variation of Epidemiological Parameters Related to Nymph-to-Larva Transmission and Exposure of Small Mammals. Pathogens 9, 518. https://doi.org/10.3390/pathogens9070518

Brabec, M., Daniel, M., Malý, M., Danielová, V., Kříž, B., Kott, I., Beneš, Č., 2017. Analysis of meteorological effects on the incidence of tick-borne encephalitis in the Czech Republic over a thirty-year period. Virol. Res. Rev. 1. https://doi.org/10.15761/VRR.1000103

Brugger, K., Boehnke, D., Petney, T., Dobler, G., Pfeffer, M., Silaghi, C., Schaub, G.A., Pinior, B., Dautel, H., Kahl, O., Pfister, K., Süss, J., Rubel, F., 2016. A Density Map of the Tick-Borne Encephalitis and Lyme Borreliosis Vector Ixodes ricinus (Acari: Ixodidae) for Germany. J. Med. Entomol. 53, 1292–1302. https://doi.org/10.1093/jme/tjw116

Brugger, K., Walter, M., Chitimia-Dobler, L., Dobler, G., Rubel, F., 2018. Forecasting next season's Ixodes ricinus nymphal density: the example of southern Germany 2018. Exp. Appl. Acarol. 75, 281–288. https://doi.org/10.1007/s10493-018-0267-6

Brugger, K., Walter, M., Chitimia-Dobler, L., Dobler, G., Rubel, F., 2017. Seasonal cycles of the TBE and Lyme borreliosis vector Ixodes ricinus modelled with time-lagged and interval-averaged predictors. Exp. Appl. Acarol. 73, 439–450. https://doi.org/10.1007/s10493-017-0197-8

Burri, C., Bastic, V., Maeder, G., Patalas, E., Gern, L., 2011. Microclimate and the Zoonotic Cycle of Tick-Borne Encephalitis Virus in Switzerland. J. Med. Entomol. 48, 615–627. https://doi.org/10.1603/ME10180

Cagnacci, F., Bolzoni, L., Rosà, R., Carpi, G., Hauffe, H.C., Valent, M., Tagliapietra, V., Kazimirova, M., Koci, J., Stanko, M., Lukan, M., Henttonen, H., Rizzoli, A., 2012. Effects of deer density on tick infestation of rodents and the hazard of tick-borne encephalitis. I: Empirical assessment. Int. J. Parasitol. 42, 365–372. https://doi.org/10.1016/j.ijpara.2012.02.012

Carpi, G., Cagnacci, F., Neteler, M., Rizzoli, A., 2008. Tick infestation on roe deer in relation to geographic and remotely sensed climatic variables in a tick-borne encephalitis endemic area. Epidemiol. Infect. 136, 1416–1424. https://doi.org/10.1017/S0950268807000039

Cuber, P., Andreassen, Å., Vainio, K., Asman, M., Dudman, S., Szilman, P., Szilman, E., Ottesen, P., Ånestad, G., Cieśla-Nobis, S., Solarz, K., 2015. Risk of exposure to ticks (Ixodidae) and the prevalence of tick-borne encephalitis virus (TBEV) in ticks in Southern Poland. Ticks Tick-Borne Dis. 6, 356–363. https://doi.org/10.1016/j.ttbdis.2015.02.010

Daniel, M., Danielová, V., Fialová, A., Malý, M., Kříž, B., Nuttall, P.A., 2018. Increased Relative Risk of Tick-Borne Encephalitis in Warmer Weather. Front. Cell. Infect. Microbiol. 8, 90. https://doi.org/10.3389/fcimb.2018.00090

Daniel, Milan, Křízˇ, B., Valter, J., Kott, I., Danielová, V., 2008. The influence of meteorological conditions of the preceding winter on the incidences of tick-borne encephalitis and Lyme borreliosis in the Czech Republic. Int. J. Med. Microbiol. 298, 60–67. https://doi.org/10.1016/j.ijmm.2008.05.001

Daniel, M., Kříž, B., Danielová, V., Valter, J., Kott, I., 2008. Correlation between meteorological factors and tick-borne encephalitis incidence in the Czech Republic. Parasitol. Res. 103, 97–107. https://doi.org/10.1007/s00436-008-1061-x

Daniel, M., Materna, J., Hönig, V., Metelka, L., Danielová, V., Harčarik, J., Kliegrová, S., Grubhoffer, L., 2009. Vertical Distribution of the Tick Ixodes ricinus and Tick-borne Pathogens in the Northern Moravian Mountains Correlated with Climate Warming (Jeseníky Mts., Czech Republic). Cent. Eur. J. Public Health 17, 139–145. https://doi.org/10.21101/cejph.a3550

Daniel, M., Vráblík, T., Valter, J., Kříž, B., Danielová, V., 2010. The TICKPRO Computer Program for Predicting Ixodes ricinus Host-seeking Activity and the Warning System Published on Websites. Cent. Eur. J. Public Health 18, 230–236. https://doi.org/10.21101/cejph.a3620

Daniel, M., Zitek, K., Danielová, V., Kříž, B., Valter, J., Kott, I., 2006. Risk assessment and prediction of Ixodes ricinus tick questing activity and human tick-borne encephalitis infection in space and time in the Czech Republic. Int. J. Med. Microbiol. 296, 41–47. https://doi.org/10.1016/j.ijmm.2006.02.008

Danielová, V., Kliegrová, S., Daniel, M., Beneš, Č., 2008a. Influence of Climate Warming on Tick-borne Encephalitis Expansion to Higher Altitudes during the Last Decade (1997-2006) in the Highland Region (Czech Republic). Cent. Eur. J. Public Health 16, 4–11. https://doi.org/10.21101/cejph.a3460

Danielová, V., Schwarzová, L., Materna, J., Daniel, M., Metelka, L., Holubová, J., Kříž, B., 2008b. Tick-borne encephalitis virus expansion to higher altitudes correlated with climate warming. Int. J. Med. Microbiol. 298, 68–72. https://doi.org/10.1016/j.ijmm.2008.02.005

Domşa, C., Mihalca, A., Sándor, A., 2018. Modeling the distribution of Ixodes ricinus in Romania. North-West. J. Zool. 14.

Dub, T., Ollgren, J., Huusko, S., Uusitalo, R., Siljander, M., Vapalahti, O., Sane, J., 2020. Game Animal Density, Climate, and Tick-Borne Encephalitis in Finland, 2007–2017. Emerg. Infect. Dis. 26, 2899–2906. https://doi.org/10.3201/eid2612.191282

Gethmann, J., Hoffmann, B., Kasbohm, E., Süss, J., Habedank, B., Conraths, F.J., Beer, M., Klaus, C., 2020. Research paper on abiotic factors and their influence on Ixodes ricinus activity—observations over a two-year period at several tick collection sites in Germany. Parasitol. Res. 119, 1455–1466. https://doi.org/10.1007/s00436-020-06666-8

Hönig, V., Svec, P., Halas, P., Vavruskova, Z., Tykalova, H., Kilian, P., Vetiskova, V., Dornakova, V., Sterbova, J., Simonova, Z., Erhart, J., Sterba, J., Golovchenko, M., Rudenko, N., Grubhoffer, L., 2015. Ticks and tick-borne pathogens in South Bohemia (Czech Republic) – Spatial variability in Ixodes ricinus abundance, Borrelia burgdorferi and tick-borne encephalitis virus prevalence. Ticks Tick-Borne Dis. 6, 559–567. https://doi.org/10.1016/j.ttbdis.2015.04.010

Hönig, V., Švec, P., Marek, L., Mrkvička, T., Dana, Z., Wittmann, M., Masař, O., Szturcová, D., Růžek, D., Pfister, K., Grubhoffer, L., 2019. Model of Risk of Exposure to Lyme Borreliosis and Tick-Borne Encephalitis Virus-Infected Ticks in the Border Area of the Czech Republic (South Bohemia) and Germany (Lower Bavaria and Upper Palatinate). Int. J. Environ. Res. Public. Health 16, 1173. https://doi.org/10.3390/ijerph16071173

Jaenson, T.G.T., Petersson, E.H., Jaenson, D.G.E., Kindberg, J., Pettersson, J.H.-O., Hjertqvist, M., Medlock, J.M., Bengtsson, H., 2018. The importance of wildlife in the ecology and epidemiology of the TBE virus in Sweden: incidence of human TBE correlates with abundance of deer and hares. Parasit. Vectors 11, 477. https://doi.org/10.1186/s13071-018-3057-4

Kiffner, C., Vor, T., Hagedorn, P., Niedrig, M., Rühe, F., 2012. Determinants of tick-borne encephalitis virus antibody presence in roe deer (Capreolus capreolus) sera. Med. Vet. Entomol. 26, 18–25. https://doi.org/10.1111/j.1365-2915.2011.00961.x

Kiffner, C., Vor, T., Hagedorn, P., Niedrig, M., Rühe, F., 2011. Factors affecting patterns of tick parasitism on forest rodents in tick-borne encephalitis risk areas, Germany. Parasitol. Res. 108, 323–335. https://doi.org/10.1007/s00436-010-2065-x

Kiffner, C., Zucchini, W., Schomaker, P., Vor, T., Hagedorn, P., Niedrig, M., Rühe, F., 2010. Determinants of tick-borne encephalitis in counties of southern Germany, 2001-2008. Int. J. Health Geogr. 9, 42. https://doi.org/10.1186/1476-072X-9-42

Kjær, L.J., Soleng, A., Edgar, K.S., Lindstedt, H.E.H., Paulsen, K.M., Andreassen, Å.K., Korslund, L., Kjelland, V., Slettan, A., Stuen, S., Kjellander, P., Christensson, M., Teräväinen, M., Baum, A., Klitgaard, K., Bødker, R., 2019. Predicting and mapping human risk of exposure to Ixodes ricinus nymphs using climatic and environmental data, Denmark, Norway and Sweden, 2016. Eurosurveillance 24. https://doi.org/10.2807/1560-7917.ES.2019.24.9.1800101

Knap, N., Avšič-Županc, T., 2015. Factors affecting the ecology of tick-borne encephalitis in Slovenia. Epidemiol. Infect. 143, 2059–2067. https://doi.org/10.1017/S0950268815000485

Knap, N., Avšič-Županc, T., 2013. Correlation of TBE Incidence with Red Deer and Roe Deer Abundance in Slovenia. PLoS ONE 8, e66380. https://doi.org/10.1371/journal.pone.0066380

Knap, N., Durmiši, E., Saksida, A., Korva, M., Petrovec, M., Avšič-Županc, T., 2009. Influence of climatic factors on dynamics of questing Ixodes ricinus ticks in Slovenia. Vet. Parasitol. 164, 275–281. https://doi.org/10.1016/j.vetpar.2009.06.001

Kolář, J., Potůčková, M., Štefanová, E., 2016. Tick-born encephalitis risk assessment based on satellite data. AUC Geogr. 51, 155–167. https://doi.org/10.14712/23361980.2016.13

Kriz, B., Daniel, M., Benes, C., Maly, M., 2014. The Role of Game (Wild Boar and Roe Deer) in the Spread of Tick-Borne Encephalitis in the Czech Republic. Vector-Borne Zoonotic Dis. 14, 801–807. https://doi.org/10.1089/vbz.2013.1569

Nah, K., Bede-Fazekas, Á., Trájer, A.J., Wu, J., 2020. The potential impact of climate change on the transmission risk of tick-borne encephalitis in Hungary. BMC Infect. Dis. 20, 34. https://doi.org/10.1186/s12879-019-4734-4

Palo, R.T., 2014. Tick-Borne Encephalitis Transmission Risk: Its Dependence on Host Population Dynamics and

Climate Effects. Vector-Borne Zoonotic Dis. 14, 346–352. https://doi.org/10.1089/vbz.2013.1386

Porretta, D., Mastrantonio, V., Amendolia, S., Gaiarsa, S., Epis, S., Genchi, C., Bandi, C., Otranto, D., Urbanelli, S., 2013. Effects of global changes on the climatic niche of the tick Ixodes ricinus inferred by species distribution modelling. Parasit. Vectors 6, 271. https://doi.org/10.1186/1756-3305-6-271

Rácz, G.R., Bán, E., Ferenczi, E., Berencsi, G., 2006. A Simple Spatial Model to Explain the Distribution of Human Tick-Borne Encephalitis Cases in Hungary. Vector-Borne Zoonotic Dis. 6, 369–378. https://doi.org/10.1089/vbz.2006.6.369

Randolph, S.E., Asokliene, L., Avsic-Zupanc, T., Bormane, A., Burri, C., Gern, L., Golovljova, I., Hubalek, Z., Knap, N., Kondrusik, M., Kupca, A., Pejcoch, M., Vasilenko, V., Zygutiene, M., 2008. Variable spikes in tick-borne encephalitis incidence in 2006 independent of variable tick abundance but related to weather. Parasit. Vectors 1, 44. https://doi.org/10.1186/1756-3305-1-44

Randolph, S.E., Rogers, D.J., 2000. Fragile transmission cycles of tick-borne encephalitis virus may be disrupted by predicted climate change. Proc. R. Soc. Lond. B Biol. Sci. 267, 1741–1744. https://doi.org/10.1098/rspb.2000.1204

Rizzoli, A., Hauffe, H.C., Tagliapietra, V., Neteler, M., Rosà, R., 2009. Forest Structure and Roe Deer Abundance Predict Tick-Borne Encephalitis Risk in Italy. PLoS ONE 4, e4336. https://doi.org/10.1371/journal.pone.0004336

Rosà, R., Andreo, V., Tagliapietra, V., Baráková, I., Arnoldi, D., Hauffe, H., Manica, M., Rosso, F., Blaňarová, L., Bona, M., Derdáková, M., Hamšíková, Z., Kazimírová, M., Kraljik, J., Kocianová, E., Mahríková, L., Minichová, L., Mošanský, L., Slovák, M., Stanko, M., Špitalská, E., Ducheyne, E., Neteler, M., Hubálek, Z., Rudolf, I., Venclikova, K., Silaghi, C., Overzier, E., Farkas, R., Földvári, G., Hornok, S., Takács, N., Rizzoli, A., 2018. Effect of Climate and Land Use on the Spatio-Temporal Variability of Tick-Borne Bacteria in Europe. Int. J. Environ. Res. Public. Health 15, 732. https://doi.org/10.3390/ijerph15040732

Rosà, R., Pugliese, A., Ghosh, M., Perkins, S.E., Rizzoli, A., 2007. Temporal Variation of *Ixodes ricinus* Intensity on the Rodent Host *Apodemus flavicollis* in Relation to Local Climate and Host Dynamics. Vector-Borne Zoonotic Dis. 7, 285–295. https://doi.org/10.1089/vbz.2006.0607

Rosà, R., Tagliapietra, V., Manica, M., Arnoldi, D., Hauffe, H.C., Rossi, C., Rosso, F., Henttonen, H., Rizzoli, A., 2019. Changes in host densities and co-feeding pattern efficiently predict tick-borne encephalitis hazard in an endemic focus in northern Italy. Int. J. Parasitol. 49, 779–787. https://doi.org/10.1016/j.ijpara.2019.05.006

Rubel, F., Brugger, K., 2021. Operational TBE incidence forecasts for Austria, Germany, and Switzerland 2019–2021. Ticks Tick-Borne Dis. 12, 101579. https://doi.org/10.1016/j.ttbdis.2020.101579

Rubel, F., Brugger, K., 2020. Tick-borne encephalitis incidence forecasts for Austria, Germany, and Switzerland. Ticks Tick-Borne Dis. 11, 101437. https://doi.org/10.1016/j.ttbdis.2020.101437

Rubel, F., Walter, M., Vogelgesang, J.R., Brugger, K., 2020. Tick-borne encephalitis (TBE) cases are not random: explaining trend, low- and high-frequency oscillations based on the Austrian TBE time series. BMC Infect. Dis. 20, 448. https://doi.org/10.1186/s12879-020-05156-7

Stefanoff, P., Rosinska, M., Samuels, S., White, D.J., Morse, D.L., Randolph, S.E., 2012. A National Case-Control Study Identifies Human Socio-Economic Status and Activities as Risk Factors for Tick-Borne Encephalitis in Poland. PLoS ONE 7, e45511. https://doi.org/10.1371/journal.pone.0045511

Stefanoff, P., Rubikowska, B., Bratkowski, J., Ustrnul, Z., Vanwambeke, S., Rosinska, M., 2018. A Predictive Model Has Identified Tick-Borne Encephalitis High-Risk Areas in Regions Where No Cases Were Reported Previously, Poland, 1999–2012. Int. J. Environ. Res. Public. Health 15, 677. https://doi.org/10.3390/ijerph15040677

Sumilo, D., Asokliene, L., Bormane, A., Vasilenko, V., Golovljova, I., Randolph, S.E., 2007. Climate Change Cannot Explain the Upsurge of Tick-Borne Encephalitis in the Baltics. PLoS ONE 2, e500. https://doi.org/10.1371/journal.pone.0000500

Švec, P., Hönig, V., Zubriková, D., Wittmann, M., Pfister, K., Grubhoffer, L., 2019. The use of multi-criteria evaluation for the selection of study plots for monitoring of I. ricinus ticks – Example from Central Europe. Ticks Tick-Borne Dis. 10, 905–910. https://doi.org/10.1016/j.ttbdis.2019.04.014

Tkadlec, E., Václavík, T., Široký, P., 2019. Rodent Host Abundance and Climate Variability as Predictors of Tickborne Disease Risk 1 Year in Advance. Emerg. Infect. Dis. 25, 1738–1741. https://doi.org/10.3201/eid2509.190684

Uusitalo, R., Siljander, M., Dub, T., Sane, J., Sormunen, J.J., Pellikka, P., Vapalahti, O., 2020. Modelling habitat suitability for occurrence of human tick-borne encephalitis (TBE) cases in Finland. Ticks Tick-Borne Dis. 11, 101457. https://doi.org/10.1016/j.ttbdis.2020.101457

Vanwambeke, S.O., Sumilo, D., Bormane, A., Lambin, E.F., Randolph, S.E., 2010. Landscape predictors of tick-borne encephalitis in Latvia: land cover, land use, and land ownership. Vector Borne Zoonotic Dis. Larchmt. N 10, 497–506. https://doi.org/10.1089/vbz.2009.0116

Walter, M., Vogelgesang, J.R., Rubel, F., Brugger, K., 2020. Tick-Borne Encephalitis Virus and Its European Distribution in Ticks and Endothermic Mammals. Microorganisms 8, 1065. https://doi.org/10.3390/microorganisms8071065

Zeimes, C.B., Olsson, G.E., Hjertqvist, M., Vanwambeke, S.O., 2014. Shaping zoonosis risk: landscape ecology vs. landscape attractiveness for people, the case of tick-borne encephalitis in Sweden. Parasit. Vectors 7, 370. https://doi.org/10.1186/1756-3305-7-370

Zeman, P., Beneš, C., 2004. A tick-borne encephalitis ceiling in Central Europe has moved upwards during the

last 30 years: Possible impact of global warming? Int. J. Med. Microbiol. Suppl. 293, 48–54. https://doi.org/10.1016/S1433-1128(04)80008-1
Zöldi, V., Reiczigel, J., Egyed, L., 2013. Monitoring the diel activity of Ixodes ricinus ticks in Hungary over three seasons. Exp. Appl. Acarol. 61, 509–517. https://doi.org/10.1007/s10493-013-9708-4

Table S2 – Full references of the selected articles.

Correlation matrix of covariates

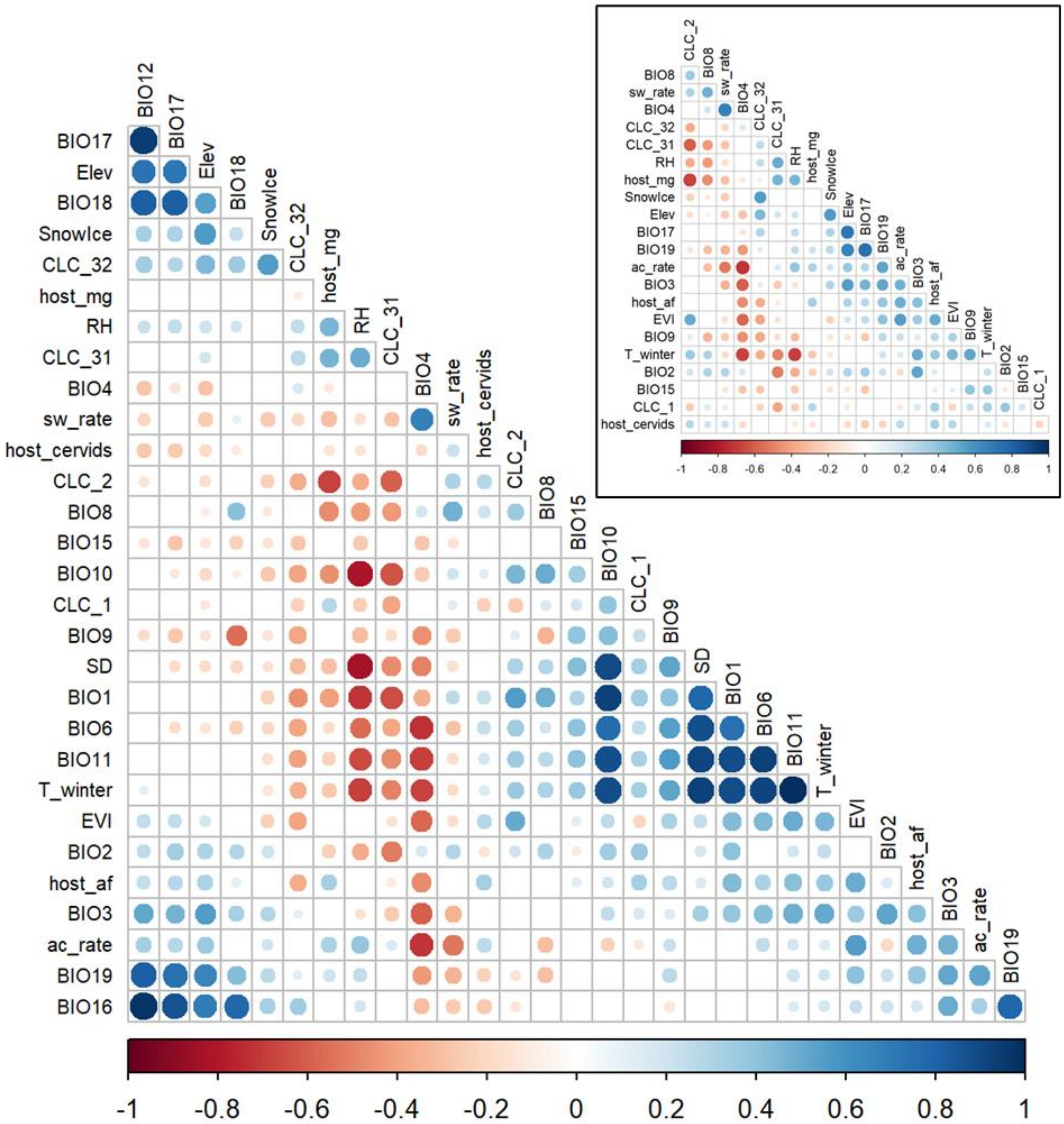

Figure S2 - Correlation matrix of covariates based on Pearson's correlation coefficient, r. Inset: correlation matrix after removing highly correlated variables (r > |0.8|). The following variables were removed at this stage: BIO1, BIO6, BIO10, BIO11, BIO12, BIO16, BIO18, SD.

Model ranking results

Model	df	logLik	AIC	ΔAIC
Y ~ CLC_31 + EVI + sw_rate + T_winter + BIO2 + BIO17 + RH + host_af + host_mg + host_cervids + host_cervids^2	14	-445·68	920·50	0
Y ~ CLC_31 + EVI + ac_rate + T_winter + BIO2 + BIO17 + host_af + host_mg + host_cervids + host_cervids^2	13	-446·77	920·53	0·03
Y ~ CLC_31 + EVI + sw_rate + T_winter + BIO2 + BIO8 + BIO17 + RH + host_af + host_mg + host_cervids + host_cervids^2	15	-444·67	920·66	0·16
Y ~ CLC_31 + EVI + ac_rate + sw_rate + T_winter + BIO2 + BIO17 + host_af + host_mg + host_cervids + host_cervids^2	14	-445·79	920·73	0·22
Y ~ CLC_31 + EVI + sw_rate + T_winter + T_winter^2 + BIO2 + BIO17 + RH + host_af + host_mg + host_cervids + host_cervids^2	15	-444·91	921·14	0·64
Y ~ CLC_31 + EVI + ac_rate + T_winter + BIO2 + BIO17 + RH + host_af + host_mg + host_cervids + host_cervids^2	14	-446·03	921·21	0·70
Y ~ CLC_31 + EVI + ac_rate + sw_rate + T_winter + BIO2 + BIO17 + RH + host_af + host_mg + host_cervids + host_cervids^2	15	-444·98	921·28	0·78
Y ~ CLC_31 + sw_rate + T_winter + BIO2 + BIO17 + RH + host_af + host_mg + host_cervids + host_cervids^2	13	-447·28	921·55	1·05
**Y ~ CLC_31 + ac_rate + T_winter + BIO2 + BIO17 + host_af + host_mg + host_cervids + host_cervids^2**	**12**	**-448·35**	**921·56**	**1·05**
Y ~ CLC_31 + EVI + sw_rate + T_winter + T_winter^2 + BIO2 + BIO8 + BIO17 + RH + host_af + host_mg + host_cervids + host_cervids^2	16	-444·07	921·64	1·14
Y ~ CLC_31 + ac_rate + sw_rate + T_winter + BIO2 + BIO17 + host_af + host_mg + host_cervids + host_cervids^2	13	-447·35	921·69	1·18
Y ~ CLC_31 + EVI + ac_rate + sw_rate + T_winter + BIO2 + BIO8 + BIO17 + RH + host_af + host_mg + host_cervids + host_cervids^2	16	-444·10	921·70	1·20
Y ~ CLC_31 + EVI + ac_rate + sw_rate + T_winter + BIO2 + BIO8 + BIO17 + host_af + host_mg + host_cervids + host_cervids^2	15	-445·27	921·86	1·35
Y ~ CLC_31 + EVI + ac_rate + T_winter + T_winter^2 + BIO2 + BIO17 + host_af + host_mg + host_cervids + host_cervids^2	14	-446·36	921·86	1·36
Y ~ CLC_31 + EVI + ac_rate + T_winter + T_winter^2 + BIO2 + BIO17 + RH + host_af + host_mg + host_cervids + host_cervids^2	15	-445·36	922·04	1·53
Y ~ CLC_31 + EVI + ac_rate + T_winter + BIO2 + BIO9 + BIO17 + host_af + host_mg + host_cervids + host_cervids^2	14	-446·45	922·05	1·54
Y ~ CLC_31 + EVI + ac_rate + sw_rate + T_winter + T_winter^2 + BIO2 + BIO17 + host_af + host_mg + host_cervids + host_cervids^2	15	-445·37	922·06	1·55
Y ~ CLC_31 + EVI + sw_rate + T_winter + BIO2 + BIO9 + BIO17 + RH + host_af + host_mg + host_cervids + host_cervids^2	15	-445·37	922·07	1·56
Y ~ CLC_31 + EVI + ac_rate + sw_rate + T_winter + T_winter^2 + BIO2 + BIO17 + RH + host_af + host_mg + host_cervids + host_cervids^2	16	-444·29	922·07	1·57
Y ~ CLC_31 + sw_rate + T_winter + BIO2 + BIO17 + host_af + host_mg + host_cervids + host_cervids^2	12	-448·65	922·15	1·65
Y ~ CLC_31 + EVI + + sw_rate + T_winter + BIO2 + BIO8 + BIO15 + BIO17 + RH + host_af + host_mg + host_cervids + host_cervids^2	16	-444·33	922·16	1·65
Y ~ CLC_31 + sw_rate + T_winter + T_winter^2 + BIO2 + BIO17 + RH + host_af + host_mg + host_cervids + host_cervids^2	14	-446·52	922·19	1·68
Y ~ CLC_31 + EVI + ac_rate + T_winter + BIO2 + BIO8 + BIO17 + host_af + host_mg + host_cervids + host_cervids^2	14	-446·55	922·24	1·74
Y ~ CLC_31 + EVI + ac_rate + T_winter + BIO2 + BIO15 + BIO17 + host_af + host_mg + host_cervids + host_cervids^2	14	-446·58	922·30	1·80
Y ~ CLC_31 + EVI + sw_rate + T_winter + BIO2 + BIO15 + BIO17 + RH + host_af + host_mg + host_cervids + host_cervids^2	15	-445·50	922·32	1·81
Y ~ CLC_31 + EVI + ac_rate + sw_rate + T_winter + BIO2 + BIO9 + BIO17 + host_af + host_mg + host_cervids + host_cervids^2	15	-445·53	922·38	1·87
Y ~ CLC_31 + sw_rate + T_winter + BIO2 + BIO8 + BIO17 + RH + host_af + host_mg + host_cervids + host_cervids^2	14	-446·62	922·38	1·88
Y ~ CLC_31 + EVI + sw_rate + T_winter + BIO2 + BIO8 + BIO17 + RH + host_af + host_mg + host_mg^2 + host_cervids + host_cervids^2	16	-444·48	922·44	1·94
Y ~ CLC_31 + EVI + sw_rate + T_winter + BIO2 + BIO17 + host_af + host_mg + host_cervids + host_cervids^2	13	-447·74	922·47	1·97

Table S3 – Top-ranked candidate models based on the Akaike's Information Criterion (AIC). df: degrees of freedom. logLik: log-likelihood. ΔAIC: delta with respect to lowest AIC score. The following variables were dropped based on their VIFs: BIO3, BIO4, BIO19, CLC_1, CLC_2, CLC_32, SnowIce. The final model selected is highlighted in bold.
